# Cytochrome P450 1A1 is essential for the microbial metabolite, Urolithin A-mediated protection against colitis

**DOI:** 10.3389/fimmu.2022.1004603

**Published:** 2022-09-08

**Authors:** Sweta Ghosh, Bhagavatula Moorthy, Bodduluri Haribabu, Venkatakrishna Rao Jala

**Affiliations:** ^1^ Department of Microbiology and Immunology, Brown Cancer Center, Center for Microbiomics, Inflammation and Pathogenicity, University of Louisville, Louisville, KY, United States; ^2^ Department of Pediatrics and Neonatology, Baylor College of Medicine, Houston, TX, United States

**Keywords:** cytochrome P450 1A1, Urolithin A, microbial metabolite, inflammatory bowel disease, junctional proteins, inflammation, immune cells, gut barrier function

## Abstract

**Background:**

Cytochrome P450 Family 1 Subfamily A Member 1 (CYP1A1) pathway, which is regulated by aryl hydrocarbon receptor (AhR) plays an important role in chemical carcinogenesis and xenobiotic metabolism. Recently, we demonstrated that the microbial metabolite Urolithin A (UroA) mitigates colitis through its gut barrier protective and anti-inflammatory activities in an AhR-dependent manner. Here, we explored role of CYP1A1 in UroA-mediated gut barrier and immune functions in regulation of inflammatory bowel disease (IBD).

**Methods:**

To determine the role of CYP1A1 in UroA-mediated protectives activities against colitis, we subjected C57BL/6 mice and *Cyp1a1* ^-/-^ mice to dextran sodium sulphate (DSS)-induced acute colitis model. The phenotypes of the mice were characterized by determining loss of body weight, intestinal permeability, systemic and colonic inflammation. Further, we evaluated the impact of UroA on regulation of immune cell populations by flow cytometry and confocal imaging using both *in vivo* and *ex vivo* model systems.

**Results:**

UroA treatment mitigated DSS-induced acute colitis in the wildtype mice. However, UroA-failed to protect *Cyp1a1* ^-/-^ mice against colitis, as evident from non-recovery of body weight loss, shortened colon lengths and colon weight/length ratios. Further, UroA failed to reduce DSS-induced inflammation, intestinal permeability and upregulate tight junction proteins in *Cyp1a1* ^-/-^ mice. Interestingly, UroA induced the expansion of T-reg cells in a CYP1A1-dependent manner both *in vivo* and *ex vivo* models.

**Conclusion:**

Our results suggest that CYP1A1 expression is essential for UroA-mediated enhanced gut barrier functions and protective activities against colitis. We postulate that CYP1A1 plays critical and yet unknown functions beyond xenobiotic metabolism in the regulation of gut epithelial integrity and immune systems to maintain gut homeostasis in IBD pathogenesis.

## Introduction

Inflammatory bowel disease (IBD) comprises of Crohn’s disease (CD) and ulcerative colitis (UC) and exhibits increased intestinal inflammation, gut epithelial barrier damage and microbial dysbiosis (1, 2). Systemic dysregulation of the immune system leading to the mucosal damage and increased gut permeability are common features of IBD (3). Microbiota and their metabolites have been shown to regulate the IBD pathogenesis by modulating major physiological processes such as immunoregulation, metabolism and gut barrier functions (2, 4–7). The molecular and cellular mechanisms of microbial metabolites in IBD pathogenesis are still emerging.

Urolithin A (UroA) is a microbial metabolite derived from polyphenolic compounds such as ellagitannins (ETs) and ellagic acid (EA) of berries and pomegranates. Urolithin A has been shown to render numerous health benefits by mediating anti-oxidative, anti- inflammatory, anti-diabetic, anti-cancer and anti-ageing effects (8–11). Previously, our group demonstrated that UroA enhanced gut barrier function through upregulating junctional proteins and reducing unwarranted inflammation through activation of aryl hydrocarbon receptor (AhR)-nuclear factor erythroid 2–related factor 2 (Nrf2)-dependent pathways (11). Further, we showed that treatment with UroA attenuated 2,4,6-trinitrobenzene sulfonic acid (TNBS) or dextran sodium sulfate (DSS)-induced colitis in mouse models (11). While UroA has been shown to activate AhR and enhance gut barrier function, involvement of downstream AhR signaling pathways that are potentially responsible for anti-inflammatory, gut barrier protective and anti-colitis activities have not been investigated.

AhR, a nuclear transcription factor, is an environmental sensor and activated by numerous xenobiotics, dietary and microbial metabolites (12–15). It is well-established that activation of AhR induces cytochrome P450 Family 1 Subfamily A Member 1 (CYP1A1) leading to metabolism of endogenous and exogenous substances including xenobiotics to excrete out mostly through Phase 1 reactions. The CYP enzymes are linked to wide variety of reactions including O-dealkylation, S-oxidation, epoxidation, and hydroxylation (16, 17). CYP enzymes are expressed in abundant levels in intestinal and hepatic tissues in humans and other organisms (18). CYP enzymes are implicated in tissue growth, development and ontogeny and are associated with pronounced changes in the microbiota (19, 20). It was shown that tremendous metabolic activity of microbiota is associated with higher levels of bacterial CYP enzymes (19–21). Activity and expression of CYP enzymes are significantly affected by microbiota composition, infections and inflammation, especially by inflammatory cytokines (18, 20, 22). However, the direct role of CYP1A1 beyond drug metabolism and its role in intestinal disorders such as in colitis are yet to be explored. Previously, it was shown that overexpression of CYP1A1 in gut epithelial cells led to decreased AhR ligands (quasi AhR-deficient) and displayed enhanced susceptibility to *Citrobacter rodentium-*induced colitis (23). Interestingly, Klotz et al. reported that CYP1A1 was significantly upregulated in ulcerative colitis patients (24). They further showed that CYP1A1 staining was positive significantly more often in patients with CD (39.6%) and UC (39.4%) than in subjects in the control group (19.2%). It is possible that increased expression of CYP1A1 potentially results in decreased availability of AhR ligands to host. Thus far, the requirement of basal level of CYP1A1 for AhR-mediated protective activities against colitis has not been reported. Additionally, the role of CYP1A1 in regulation of microbial metabolite-mediated activities other than drug metabolism are yet to be established.

In the current study, we examined the role of CYP1A1 in UroA-mediated protective activities against colitis (14). Our current study suggests that CYP1A1 is critical for UroA-mediated regulation of intestinal barrier function, anti-inflammatory activities and mitigation of colitis. Further, this study unraveled that UroA treatment enhances the expansion of T-reg cells without effecting Th17 cell proliferation in CYP1A1 dependent manner. Overall, here for the first time, we showed that UroA requires the functional expression of CYP1A1 to exert its physiological activities and mitigation of colitis in pre-clinical models.

## Materials and methods

### Reagents and chemicals

General laboratory chemicals and reagent solutions were procured either from VWR (Radnor, PA) or Sigma-Aldrich (St. Louis, MO) unless otherwise specified. ELISA kits for IL-6 and TNF-α were purchased from Bio-legend (San Diego, CA). UroA was custom synthesized as previously described (10, 11). Colitis grade DSS (36,000–50,000 M.W) was purchased from MP Biomedicals (Irvine, CA). Antibodies for flow cytometry were purchased from Bio-legend. Western blot antibodies were purchased either from Santacruz (Dallas, TX) or ProteinTech (Rosemont, IL). The complete list of antibodies is provided in **Supplementary Tables 1, 2**. Reagents for Alcian Blue-Periodic acid–Schiff (AB-PAS) staining were obtained from electron microscopy sciences (Hatfield, PA).

### Mice

Wildtype (C57BL/6) mice were generated at U of L animal facility. Breeding pairs of *Cyp1a1* ^-/-^ mice were kind gift from Dr. Bhagavatula Moorthy (Baylor College of Medicine, Houston, TX, USA) after obtaining MTA from University of Cincinnati, USA. *Cyp1a1* ^-/-^ mice were bred at our animal facility to generate experimental animals. 6–8 weeks age old mice were utilized for DSS-induced acute colitis experiments. Mice were kept with alternate 12 h cycles of dark and light in a temperature-controlled and specific pathogen-free (SPF) barrier conditions. Mice were provided with food and water ad libitum. All the experiments were performed under approved protocols from Institutional Animal Care and Use Committee (IACUC), University of Louisville, Louisville, KY, USA.

### DSS-induced colitis

Mice received 2.5% DSS (36,000–50,000 MW, MP Biomedicals) in their drinking water for 7 days, followed by 5 days of normal water without DSS. Control animals received normal water for the entire period. Mice were monitored daily for body weight and sacrificed on day 12 post DSS. All colitis group mice were randomly divided into groups and received the oral treatment of either vehicle (1% CMC and 0.1% Polysorbate 80) or UroA (20 mg/kg) on the alternate day starting from day 2 of DSS treatment. UroA was prepared in 1% CMC and 0.1% Polysorbate 80.

### Assessment of colitis severity and tissue collection

Experimental mice were examined daily for changes in body weight, rectal bleeding, and stool texture. After euthanasia, mice blood was collected by cardiac puncture method. The colons were removed and the gross images were captured. The colons were flushed with 1XPBS followed by measurement of colon length and colon weight. Parts of colon were either snap frozen in liquid nitrogen and stored in −80 °C or were stored in 10% phosphate buffered saline formalin until further analysis. Mice spleen and mesenteric lymph nodes (mLN) were collected in complete RPMI medium (containing 10% fetal bovine serum (FBS, Gibco) and 1X penicillin-streptomycin solution (100 U/ml penicillin, and 100 µg/ml streptomycin).

### Histopathology

Colon tissues from mice were fixed with 10% formalin solution overnight followed by stored in 70% alcohol. After fixation, tissues were processed with standard histopathological processing for paraffin embedding, and paraffin sections of 5μm were cut by Horus Scientific (MA, USA). The tissue sections were processed and Haemotoxylin and Eosin (H&E) staining was performed. H&E images were captured using PanDesk Slide Scanner (3DHISTECH Ltd., MI, USA). For histological staining of mucin, Alcian Blue-Periodic acid–Schiff (AB-PAS) staining was performed on the 5μm paraffin sections of colon tissues. The colon sections were deparaffinized and hydrated to distilled water using a standard protocol. Colon sections were then stained with Alcian blue (pH 2.5) (1% in 3% acetic acid) for 30 min and the slides were rinsed with running tap water for 2 min. Sections were then incubated with 0.5% aqueous solution of Periodic Acid for 10 minutes. After slides were rinsed in running tap water for 5 min, tissues were stained with Schiff’s reagent for 20 mins. Slides were further rinsed in running tap water for 5 min. Tissue sections were counterstained for 5 min with 0.1% nuclear fast red solution, followed by rinsing in running tap water for 1 min. Stained tissue section were dehydrated in 100%, 95%, 90%, 70% 50% ethanol 3 min each and cleared in xylene substitute for 20 min. The tissues were mounted in xylene substitute mounting solution (Thermo-fisher scientific) with a coverslip, dried overnight and imaged using PanDesk Slide Scanner (3DHISTECH Ltd., MI, USA).

### Immunofluorescence

The paraffin section slides were permeabilized and stained with FoxP3 or F4/80 specific primary antibody (1:100 dilution) followed by Alexa flour 647 and Alexa flour 594 secondary antibody (1:500 dilution) respectively as described previously (25). Stained sections were mounted with VECTASHIELD HardSet™ antifade mounting medium with DAPI (Vector Laboratories) to stain the nuclei. The immunofluorescence images were captured using a Nikon A1R confocal microscope using appropriate laser channels.

### *In vivo* intestinal permeability determination

The gut barrier permeability was assessed by *in vivo* intestinal permeability assay using FITC-dextran (MW 4 kDa FD4, Sigma-Aldrich, USA). Briefly, FITC-dextran (60 mg/100 gm body weight) was orally administered to the mice 4 h prior to euthanization (fasted state). The blood was collected by cardiac puncture, prepared the serum. FITC-dextran levels in serum were evaluated using Synergy HT Microplate Reader (Biotek, VT, USA) at Ex, 485 nm, Em 525 nm.

### Western blotting

The colon tissues were homogenized in RIPA buffer (Sigma) with 1X protease inhibitors (Roche). Western blots for respective proteins were performed as previously described (11). The Western blots were probed for either horseradish peroxidase (HRP) conjugated Cldn-4 and β-actin antibodies or ZO-1 and Occludin antibodies followed by rabbit secondary antibody conjugated with HRP. The protein bands were detected with chemiluminescent substrate and imaged using a Biorad ChemiDoc Imaging System (Hercules, CA). Densitometry analysis of bands were done using ImageJ software. List of the antibodies with catalogue number and working dilutions are provided in **Supplementary Table 1**.

### Quantitative real-time polymerase chain reaction (qRT-PCR)

Total RNA was prepared from colon tissues of WT and *Cyp1a1^−/−^* mice using Maxwell^®^ 16 LEV simplyRNA tissue kits (Promega) and changes in expression of ZO-1, Occludin and Cldn4 genes were analyzed as previously described (9, 11, 26). Fold changes in gene expression levels were calculated using the 2^-ΔΔ^CT method using β-actin as a housekeeping gene control and normalized to the untreated control of each mice group.

### Myeloperoxidase (MPO) activity

MPO activity of mice colon tissue was determined using Myeloperoxidase (MPO) Activity Assay Kit (Abcam) as per manufactures protocol and readings were taken (OD at 412 nm) using a Synergy HT Microplate Reader (Biotek, VT, USA).

### Isolation of lymphocytes and flow cytometry

Cells from mesenteric lymph nodes (mLNs) of WT and CYP1A1^−/−^ mice were isolated and stained for macrophages, neutrophils, dendritic cells and T cells using standard flow cytometric methods as described elsewhere (25, 27). Cells were stained with appropriate fluorochrome labeled anti-mouse CD3, CD4, CD8, CD11c, CD11b, F4/80, CD45RB, F4/80, and CD45 Ab. The flowcytometric results were acquired with using BD FACSCanto II. Data were analyzed using FlowJo software (Tree Star). List of the antibodies with catalogue number are provided in **Supplementary Table 2**. Intracellular staining was performed using True-Nuclear™ Transcription Factor Buffer Set (Biolegend, San Diego, CA, USA) as per manufacturer’s instructions. For Treg and Th17 cell analysis, single cell suspensions from the spleen or mLN were stimulated with the Cell Activation Cocktail (Biolegend) containing phorbol myristate acetate (PMA), ionomycin, and brefeldin A (Biolegend) and monensin (Biolegend), for 6 hours and stained for FoxP3 and IL17A as per manufacturer’s instructions.

### Bone marrow derived macrophages (BMDMs) preparation

Mouse BMDMs were isolated and cultured from wildtype and *Cyp1a1^−/−^* mice. Briefly, mice were euthanized, and bone marrow was procured from tibias and femurs. For differentiation, collected bone marrow cells were plated (2 × 10^6^ cell/ml) in DMEM-high glucose supplemented with 10% FBS, 1% glutamine, 1X penicillin-streptomycin solution, 50 μM β-ME and 100 ng/mL mouse M-CSF (Biolegend) for 7 days. BMDMs were plated in 96 well plate (10,000 cells/well) for ELISA. To evaluate the anti-inflammatory properties of UroA on BMDMs, the cells were stimulated with *E. coli*-derived lipopolysaccharides (LPS; O55:B5; Sigma) at 50 ng/ml concentration for six hours alone or in combination with different concentrations UroA (0, 10, 25, and 50 μM) in quadruplicates.

### Measurements of IL-6, TNF-α and IL-1β levels

Serum cytokines or BMDM supernatants were quantified using mouse TNF-α, IL-6 and IL-1β specific ELISA kits (Biolegend) following manufacturer’s instruction using Synergy HT Microplate Reader (Biotek, VT, USA).

### *In vitro* T cell differentiation

Single cell suspensions from the spleen or mLN were prepared from wildtype and *Cyp1a1^−/−^* mice and the cells were resuspended at 1 × 10^6^/mL in complete medium (RPM1-1640 containing 10% FBS, L-glutamine, 1X penicillin/streptomycin). These cells were added to pre-coated anti-CD3e (BioLegend, San Diego, CA, USA) 96-well-plate to stimulate T cell proliferation. Cells were grown for 72 h in the complete media containing 5 μg/mL of anti-CD28 (BioLegend, San Diego, CA, USA). The cells were stained for CD4^+^ T cells using standard flow cytometric methods using BD FACSCanto II. Data were analyzed using FlowJo (Tree Star) software. For Treg and Th17 cell analysis, single cell suspensions from the spleen or mLN were stimulated with the Cell Activation Cocktail (Biolegend) containing phorbol myristate acetate (PMA), ionomycin, and brefeldin A (Biolegend) and monensin (Biolegend), for 6 hours and stained for FoxP3 and IL17A as per manufacturer’s instructions.

### Statistical analysis

Statistical analysis was performed (ANOVA followed by Tukey’s Multiple Comparisons Test with p < 0.05 taken as significant) using Graphpad Prism software (GraphPad Software, San Diego, USA) using. Specific details of the statistical tests are provided in the figure legends. Data are represented as mean ± SEM from triplicate determinations, unless otherwise specified in the figure legends.

## Results

### CYP1A1 is critical for UroA-mediated protection against 2.5% DSS induced colitis

**Figure 1:**
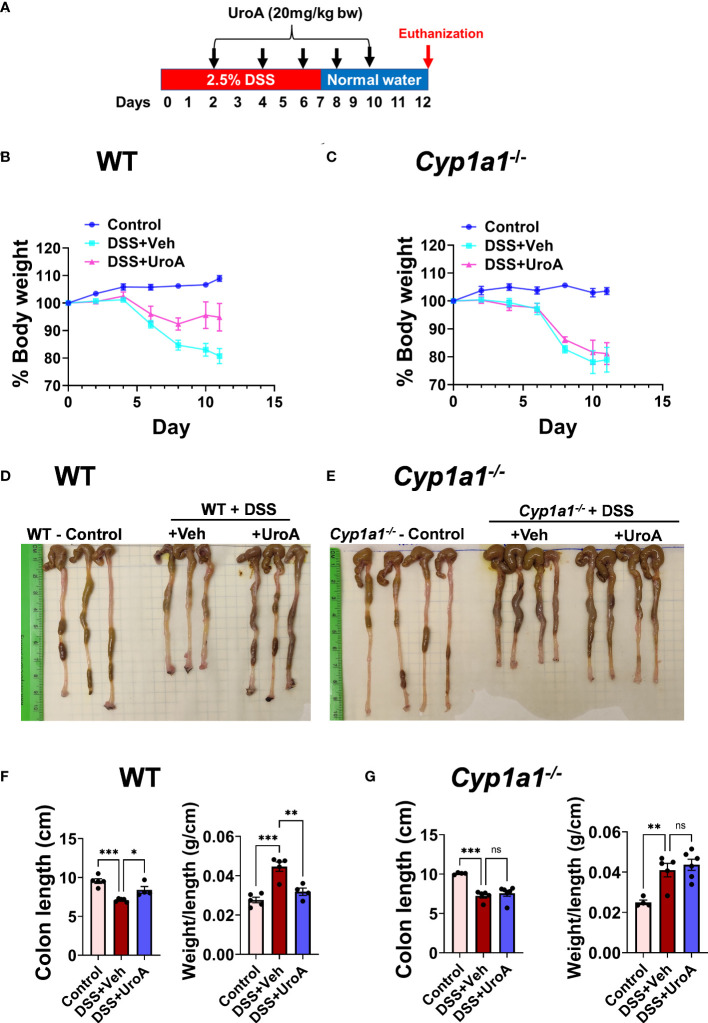
Urolithin A (UroA) mitigates DSS-induced colitis in CYP1A1-dependent manner. **(A)** Scheme of acute DSS-induced colitis model. C57BL/6 or *Cyp1a1^-/-^* mice (n=5-7, 6-8 week age old) were subjected to 2.5% DSS in drinking water for 7 days and switched back to normal water and continued for additional 5 days. Mice were euthanized at day 12. Mice were treated on alternate day with either vehicle of UroA (20 mg/kg) orally 2 days post DSS treatment. Control mice received normal water. **(B, C)**. Percent body weights of wild type (WT) **(B)** and *Cyp1a1^-/-^* mice **(C)** are shown. **(D, E)**. Representative gross images of colons of WT **(D)** and *Cyp1a1^-/-^* mice **(E)** were shown. **(F, G)**. Colon length and colon weight/length of WT mice **(F)** and *Cyp1a1^-/-^* mice **(G)** were measured. Statistics were performed by One Way ANOVA test using Graphpad Prism 9. ***p < 0.001; **p < 0.01 *p < 0.05; ns: Not significant. Error bars, ± SEM.

Previously, we showed that UroA-mediates gut barrier protective, anti-inflammatory and anti-colitis activities in an AhR-dependent manner (11). We have shown that treatment with UroA induced AhR downstream regulator CYP1A1 both *in vitro* (intestinal epithelial cells, macrophages) and *in vivo* (intestines and liver). Here, we asked whether AhR downstream inducer, CYP1A1 is required for UroA-mediated functional activities to protect against colitis. To address the role of CYP1A1 in colitis, the *Cyp1a1^−/−^* mice along wildtype (WT) mice were subjected to dextran sodium sulfate (DSS)-induced acute colitis model. DSS is chemical disrupter of gut barrier homeostasis leading to inflammation and gut epithelial tissue damage in colon (28). WT and *Cyp1a1* ^−/−^ mice received 2.5% DSS in drinking water for 7 days followed by normal water for 5 days (**Figure 1A**). Mice were euthanized at day 12 post DSS and characterized. These mice were orally treated either with vehicle (1% sodium carboxymethylcellulose, CMC and 0.1% Polysorbate 80) or UroA (20 mg/kg body weight) at 48 h intervals post DSS treatment (**Figure 1A**). Control group of mice received normal drinking water without DSS. Oral treatment with UroA protected WT mice from the DSS- induced body weight loss (**Figure 1B**) but failed to protect the *Cyp1a1* ^−/−^ mice (**Figure 1C**). Further, UroA significantly protected WT mice from DSS-induced colon shortening (**Figures 1D**, **F**), and decreased colon weight/length ratio (**Figure 1F**). However, UroA treatment failed to protect against colon shortening, weight/length ratio in *Cyp1a1^-/-^* mice (**Figures 1E, G**). These results suggest that normal expression of CYP1A1 is required for UroA-mediated protective activities against DSS-induced colitis that may not be compensated by other CYP enzymes.

### UroA treatment failed to protect against DSS-induced colon tissue damage in *Cyp1a1* ^−/−^ mice

**Figure 2:**
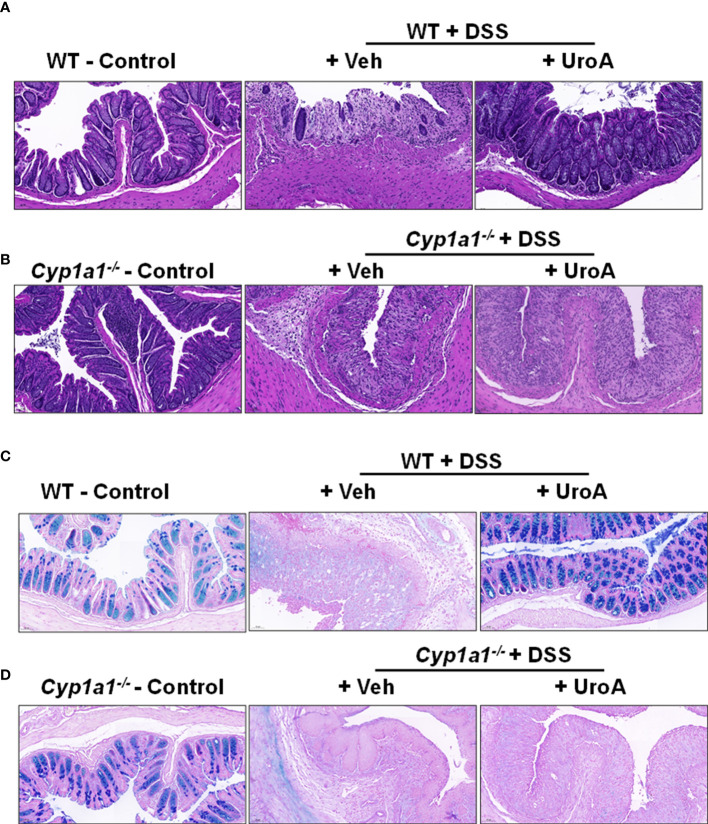
UroA treatment failed to restrict colitis related histopathological changes in *Cyp1a1^-/-^* mice. **(A, B)**. Colons of wild type (A) and *Cyp1a1^-/-^* mice (with or without DSS and UroA as described in **Figure 1**) were washed with 1X PBS and fixed with 10% formalin and processed with paraffin embedding. The donut shape sections were cut (5 μm) and stained with Hematoxylin and eosin (H&E). **(C, D)**. Alcian blue-PAS stained mucosa in sections of colons are shown. Scale bar indicates 50 μm.

The colon tissues from above-described mice were analyzed for tissue damage by histological methods. Consistent with previous findings, H&E analysis of colon sections of WT mice treated with DSS significantly damaged colon epithelial barrier. H&E analysis of colons suggested that increased infiltration of inflammatory cells, enhanced ulceration, loss of barrier architecture in DSS treated WT and *Cyp1a1* ^−/−^ mice (**Figures 2A, B**). Treatment with UroA protected the WT but not *Cyp1a1^-/-^* mice from DSS-induced tissue destruction and inflammation (**Figure 2A**). Next, we analyzed the mucin levels in the colons by alcian blue- periodic acid Schiff (AB-PAS) staining method. The AB stains the acidic mucins blue, while PAS stains the neutral mucins pink to red (29). As shown in **Figures 2C, D** (left panels), alcian blue and PAS stained mucins normally in control mice. In contrast, DSS treated mice significantly lost alcian blue and PAS staining both in colons of WT and *Cyp1a1^-/-^* mice indicating extent of intestinal damage (**Figures 2C, D**, middle panels). Importantly, treatment with UroA reversed or restored the mucin levels in WT colitis (DSS+UroA) mice but not in *Cyp1a1^-/-^* mice (**Figures 2C, D** right panels). These results indicate that UroA mediated gut protective activities (including mucin levels) require expression of CYP1A1.

### UroA-induced gut barrier enhancement is dependent on CYP1A1

**Figure 3:**
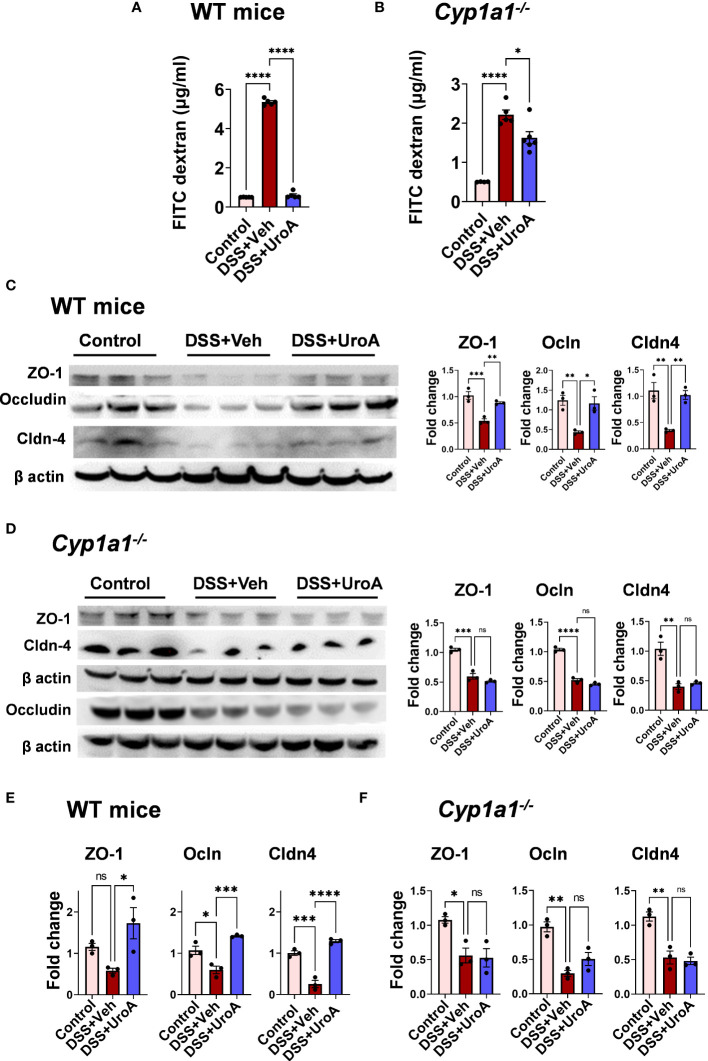
UroA-enhanced gut barrier function is dependent upon CYP1A1. Mice were subjected to acute colitis model as described in **Figure 1**. **(A, B)**. (A) Intestinal permeability of WT **(A)** and *Cyp1a1^-/-^* mice **(B)** mice was measured using FITC-dextran flux (4 KDa) assay. **(C, D)**. Expression patterns of Zonula occluden 1 (ZO-1), occludin (Ocln*)* and claudin 4 (Cldn4*)* proteins in the colons of WT mice **(C)** and *Cyp1a1^-/-^* mice **(D)** (*n* = 3) were measured by Western blot and quantified by using Image J software. **(E, F)**. The fold changes in mRNA levels of ZO1, Ocln, and Cldn4 in the colons of WT mice **(E)** and *Cyp1a1^-/-^* mice **(F)** (*n* = 4) were determined by SyBR green RT-PCR method. Statistics were performed by One Way ANOVA test using Graphpad Prism 9. ****p < 0.0001 ***p < 0.001; **p < 0.01 *p < 0.05; ns, not significant. Error bars, ± SEM.

To investigate the contribution of CYP1A1 in UroA-mediated gut barrier protective activities against DSS-induced colitis, we have evaluated the gut permeability. Intestinal permeability was determined by *in vivo* FITC-dextran (4 KDa) permeability as described previously (11). FITC-dextran was orally delivered and measured the levels of FITC-dextran in the serum to determine the extent of gut permeability. As shown in **Figure 3A**, DSS treatment increased FITC-dextran leakage in vehicle treated mice. UroA treatment significantly reduced the leakage of FITC-dextran in DSS-induced colitis mice. Interestingly, we observed that very mild reduction of permeability in *Cyp1a1^-/-^* mice (colitis mice) upon treatment of UroA compared to WT mice (**Figure 3B**). Increased permeability is associated with loss of junctional proteins in the gut epithelial cells, which are responsible for integrity of the intestinal barrier (30–32). Next, we determined the expression levels of tight junctional proteins (ZO1, Ocln, and Cldn4) in DSS treated WT and *Cyp1a1^-/-^* mice colons. As shown in **Figures 3C, D**, DSS-induced the downregulation of the tight junctional proteins (TJPs) at protein level. Treatment with UroA protected from the DSS-induced downregulation of TJPs in WT mice (**Figure 3C**), but not in colons of DSS-treated *Cyp1a1^-/-^* mice (**Figure 3D**). In agreement with protein levels, similar changes were observed at the mRNA level of TJPs in colons of DSS+veh vs DSS+UroA treated WT or *Cyp1a1^-/-^* mice (**Figures 3E, F**). Overall, these results suggest that UroA protects against DSS-induced permeability and rescues from TJP dysregulation in a CYP1A1 dependent manner.

### Anti-inflammatory activities of UroA are dependent upon CYP1A1 expression

**Figure 4:**
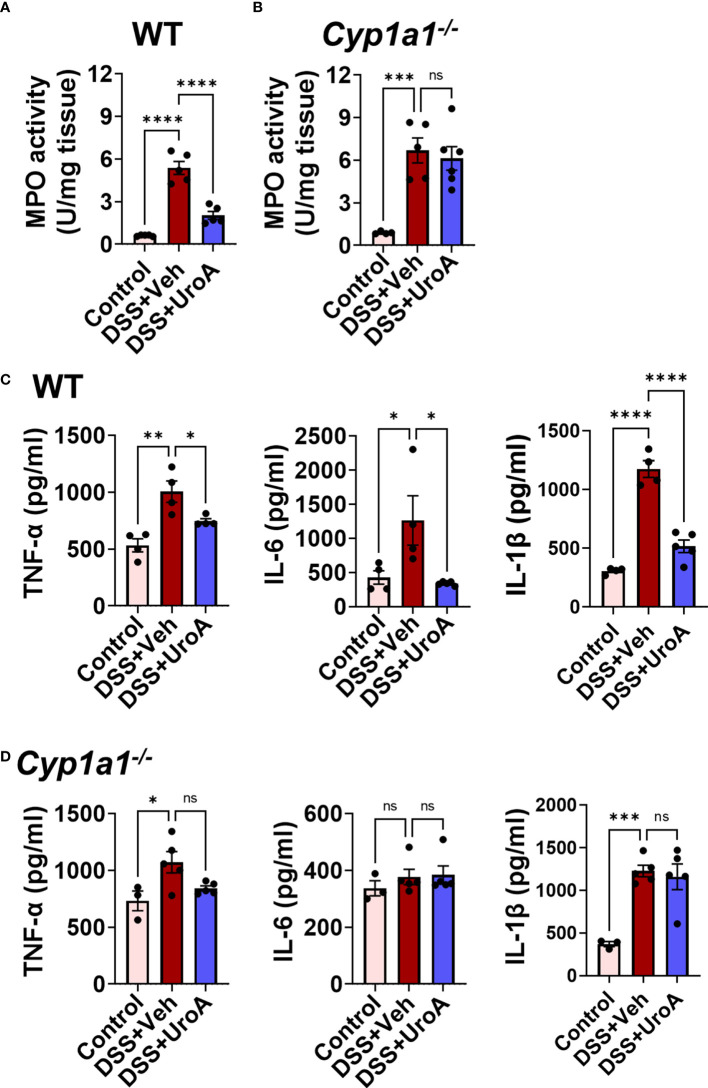
CYP1A1 is required for UroA-mediated anti-inflammatory activities. Mice were subjected to acute colitis model as described in **Figure 1** **. (A, B)**. MPO levels in colons of WT **(A)** and *Cyp1a1^-/-^* mice **(B)** were measured. **(C, D)** Serum levels of TNF-α, IL-6 and IL-1β in indicated mice were measured by standard ELISA methods. Statistics were performed by One Way ANOVA test using Graphpad Prism 9. ****p < 0.0001 ***p < 0.001; **p < 0.01 *p < 0.05; ns, not significant. Error bars, ± SEM.

IBD is associated with active inflammation with the risk of hyperactivation of immune responses (33). To assess the colonic inflammation, myeloperoxidase (MPO) activity was measured from colons of WT and *Cyp1a1^-/-^* mice that were subjected to colitis models. MPO activity represents the level of neutrophil infiltration into the colon. Treatment with UroA significantly reduced the colonic MPO in in WT mice that were subjected to colitis (**Figure 4A**). However, UroA treatment failed to block the increased MPO levels in *Cyp1a1^-/-^* mice that were subjected DSS-induced colitis model (**Figure 4B**). Serum inflammatory markers such as TNF-α, IL-6 and IL-1β are often considered as hallmark of ulcerative colitis (33). UroA treatment significantly reduced DSS-induced serum inflammatory cytokines (TNF-α, IL-6 and IL-1β) in WT mice (**Figure 4C**), but not in *Cyp1a1^-/-^* mice (**Figure 4D**).

**Figure 5:**
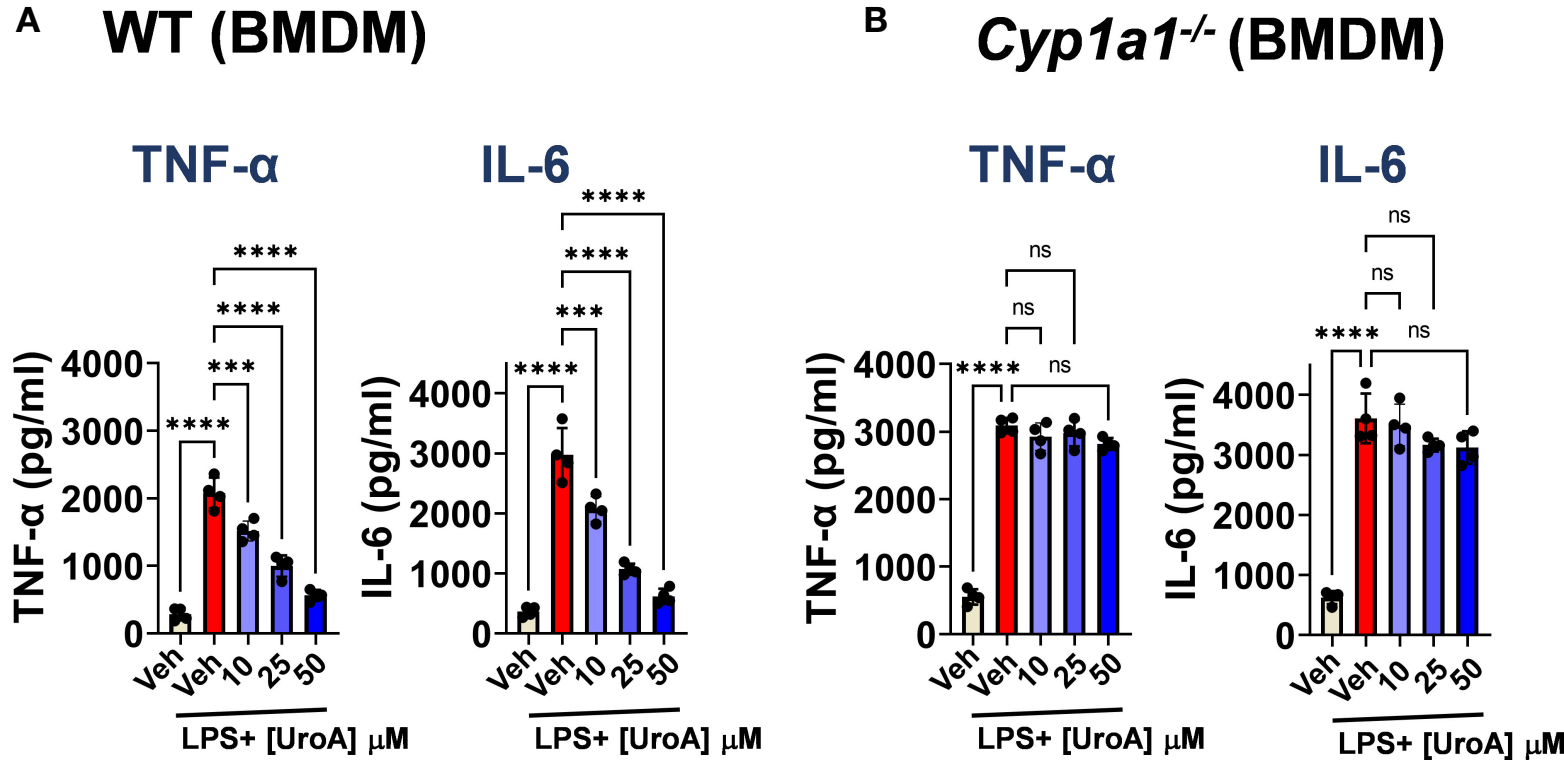
UroA reduced LPS-induced inflammatory mediators in CYP1A1-dependent manner. Bone marrow derived macrophages (BMDM) that were prepared from WT and *Cyp1a1^-/-^* mice were treated with vehicle (0.01% DMSO) or UroA in the presence or absence of LPS (50 ng/ml). The levels of TNF-α and IL-6 in the supernatants were measured using standard ELISA. Statistics were performed by One Way ANOVA test using Graphpad Prism 9. ****p < 0.0001 ***p < 0.001; ns, not significant. Error bars, ± SEM.

Next, we tested whether UroA mediated anti-inflammatory activities were dependent upon CYP1A1 using bone marrow derived macrophages (BMDMs) isolated from WT and *Cyp1a1^-/-^* mice. As shown in **Figure 5A**, UroA reduced lipopolysaccharide (LPS)-induced TNF-α and IL-6 in dose-dependent manner in WT BMDM. However, UroA failed to block LPS-induced TNF-α and IL-6 cytokines in *Cyp1a1^-/-^* BMDM (**Figure 5B**). These results suggested that UroA-mediated anti-inflammatory activities were dependent upon CYP1A1 signaling pathways.

### UroA reversed the DSS-induced immune abnormalities in CYP1A1-dependent manner

**Figure 6:**
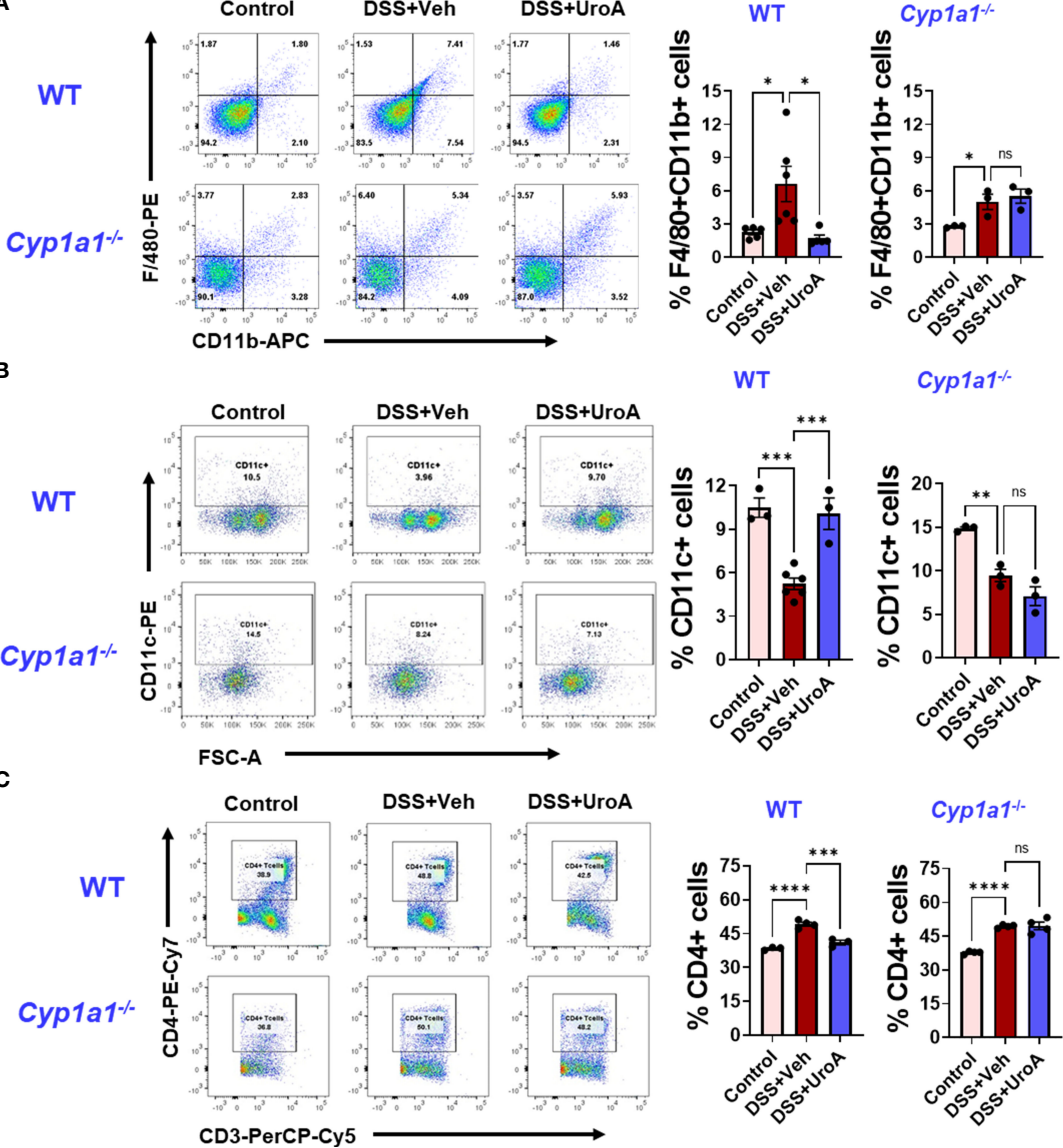
UroA fails to restore immune abnormalities in *Cyp1a1^-/-^* mice. Mice were subjected to acute colitis model as described in **Figure 1**. Immune cells from WT and *Cyp1a1^-/-^* mice were analyzed using standard flow cytometric procedures. The percentages of **(A)** F4/80^+^CD11b^+^ **(B)** CD11c^+^ **(C)** CD4^+^ T cells in the mesenteric lymph node are shown. Statistics were performed by One Way ANOVA test using Graphpad Prism 9. ****p < 0.0001 ***p < 0.001; **p < 0.01 *p < 0.05; ns, not significant. Error bars, ± SEM.

The etiology of colitis is associated with inflamed intestinal sites with inflammatory immune cells and dysregulated immune response (34). To investigate whether UroA changes the DSS-induced inflammatory immune cell balance in CYP1A1 dependent manner, we profiled the immune cells from mesenteric lymph node (mLN) of DSS-induced colitis mice, and compared with control and UroA treated mice. As expected F4/80^+^CD11b^+^ (macrophages) cells were significantly increased in mLN of DSS-treated WT and *Cyp1a1^-/-^* mice compared to control mice (**Figure 6A**). UroA treatment corrected immune abnormality by restoring to homeostatic levels of F4/80^+^CD11b^+^ cells in WT mice, but not in *Cyp1a1^-/-^* mice. Next, we analyzed the F4/80^+^ cell population in colonic tissue sections by immunofluorescence methods. As shown in **Supplementary Figure 1**, the F4/80^+^ inflammatory macrophages in colon tissues were significantly increased in DSS-treated wild type mice and *Cyp1a1^-/-^* mice compared to no-DSS treated mice. Interestingly, UroA treatment significantly reduced F4/80^+^ inflammatory macrophages in colons of wild type mice but in *Cyp1a1^-/-^* mice. These observations are similar to F/480^+^ immune cell population observed in mesenteric lymph nodes (mLN) (**Figure 6A**). Overall, these results suggested that UroA reduced the F4/80^+^ inflammatory macrophages in CYP1A1-dependent manner. There is significant decrease in the percentage of CD11c^+^ dendritic cells (DCs) population in WT and *Cyp1a1^-/-^* mice after DSS challenge (**Figure 6B**). Treatment with UroA restored the DC population in WT mice but not in *Cyp1a1^-/-^* mice that were subjected to DSS-induced colitis (**Figure 6B**). We further analyzed CD11c^+^ population with CD11b and found that the results are independent of CD11b expression (**Supplementary Figure 2**). These data suggested that the UroA-mediated modulation of inflammatory macrophages and DCs were abrogated in *Cyp1a1^-/-^* mice.

Previous reports suggested that CD4^+^ T cells were enriched in inflammatory tissues of Crohn’s and UC patients, and blockade or depletion of CD4^+^ T is effective in treating patients with IBD (35, 36). Since we observed UroA-mediated protective activities against colitis, we asked whether UroA treatment has any impact on T cell population in mLNs. As shown in **Figure 6C**, total CD4^+^ T cells were significantly increased in DSS-induced colitis mice compared to control mice. Importantly, UroA treatment significantly decreased (or restored to control level) DSS-induced CD4^+^ T cell population in mLNs of WT mice. However, UroA failed to decrease the CD4^+^ T cell population in mLNs of DSS-challenged *Cyp1a1^-/-^* mice. Next, we evaluated the effect of UroA treatment on CD8^+^ T cell population in these colitis mice (**Supplementary Figure 3**). Our results suggested that the percentage of CD8^+^ T cells (CD3^+^CD8^+^) were reduced in the mesenteric lymph node of the WT and *Cyp1a1^−/−^* mice after DSS challenge. Interestingly, UroA treatment restored the CD8^+^ T cells frequencies in WT mice, but failed in *Cyp1a1^−/−^* mice. These results indicate the CYP1A1 plays an important role in UroA-mediated immunomodulatory activities to restore the immune homeostasis.

### UroA induced T-reg cells expansion in colitis

**Figure 7:**
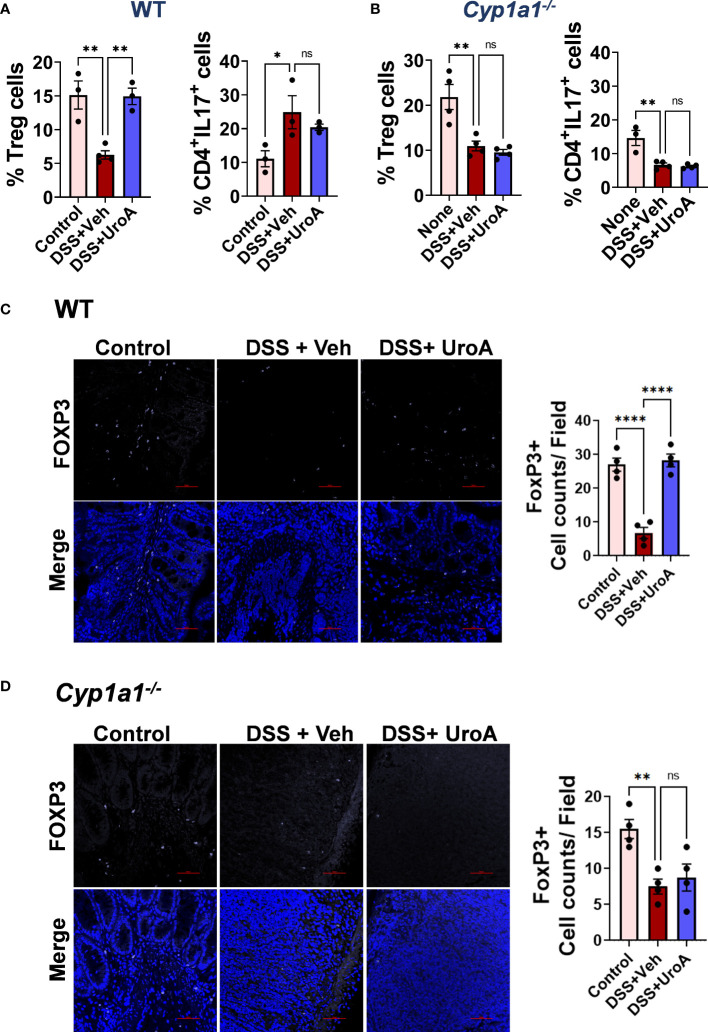
UroA treatment protected from DSS-induced depletion of T-reg cells in CYP1A1-dependent manner. Mice were subjected to acute colitis model as described in **Figure 1**. Flowcytometric analysis of T reg and Th17 cells in mesenteric lymph nodes (mNLN) of wild type (WT) **(A)** and *Cyp1a1^-/-^* **(B)** mice. Lymphocytes were first gated on a forward scatter (FSC)/side scatter (SSC) plot and single cells were then gated on the CD3^+^ population. These cells were then further gated for the subsets of interest, CD4^+^CD25^+^FOXP3^+^, T reg cells and CD4^+^IL17^+^, Th17 cells. **(C)** Colon section of WT and *Cyp1a1^-/-^* mice (Vehicle, DSS+Veh, DSS+UroA) were stained with anti- FOXP3 antibody followed by secondary antibody tagged with Alexa Fluor 647 (purple). The nucleus was stained with DAPI (blue). The fluorescence images were captured using Nikon A1R confocal microscope. The scale bar indicates 50 μm. The number of FOXP3^+^ cells per view were counted and plotted. Data are shown as mean ± SEM ****p < 0.0001; **p < 0.01; *p < 0.05.

IBD patients exhibit significant reduction in T-reg cells (37), and conversely a variety of studies suggest that increased levels of T-regs may protect against IBD pathogenesis (38–40). Previously, it was shown that activation of AhR promoted T-reg expansion in the gut (41, 42), but the role of CYP1A1 in T-reg cells expansion had not been established. Therefore, we investigated the effects of UroA on T cell differentiation. As shown in **Figures 7A, B** (left panels), T-reg cells in mLN were significantly reduced both in WT and *Cyp1a1^-/-^* mice that were subjected DSS-induced colitis. UroA treatment restored the T-reg population in WT mice, but not in *Cyp1a1^-/-^* mice (**Figures 7A, B**). UroA treatment did not alter the Th17 population in these mice (**Figures 7A, B**, right panels). Next, we examined the presence of T-reg cells in the colonic tissues of WT and *Cyp1a1^-/-^* mice that were challenged with DSS. In agreement with the mLN T-reg levels, mice challenged with DSS showed significant reduction in T-reg cell numbers compared to control mice (**Figures 7C, D**, left and middle panels). Treatment with UroA restored the T-reg population in WT mice (**Figure 7C** right panel), but not in *Cyp1a1^-/-^* mice (**Figure 7D** right panel) that were challenged with DSS.

**Figure 8:**
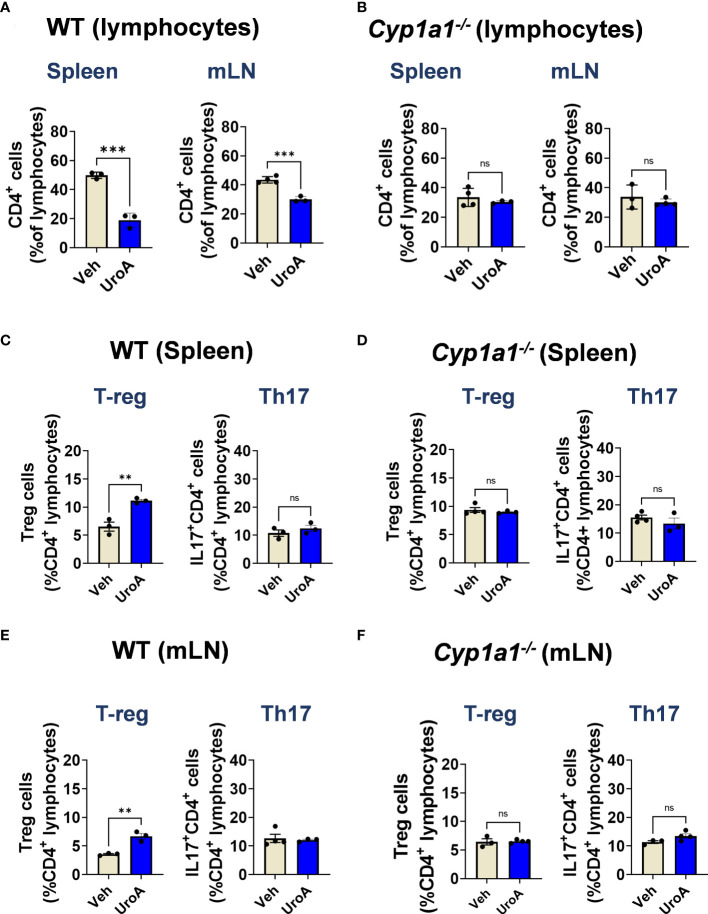
UroA induced T-reg population in CYP1A1-dependent manner. Lymphocytes from spleen and mesenteric lymph nodes (mLN) were isolated from wildtype C57BL/6 mice and *Cyp1a1^-/-^* mice (7–8 weeks old). Lymphocytes (5X10^5^ cells/well) were cultured for 3 d with anti-CD3/CD28 in the presence of vehicle (0.05% DMSO) or UroA (50 μM). **(A, B)**. Flow cytometry analysis of CD4^+^ T cells were performed and the percentages of CD4^+^ T cells of WT **(A)** and *Cyp1a1^-/-^* mice **(B)** are shown. **(C-F)**. For T reg and Th17 analysis, lymphocytes were stimulated with PMA/ionomycin or left unstimulated and brefeldin A was added. CD4+ T cells were stained for FoxP3and IL-17A. Data are representative of two independent experiments. Statistics were performed by One Way ANOVA test using Graphpad Prism 9. ****p < 0.0001 **p < 0.01; ns, not significant. Error bars, ± SEM.

To define whether UroA treatment is directly responsible for expansion of T-reg cells in CYP1A1-dependent manner, we performed *ex vivo* T cells differentiation experiments. We have utilized the lymphocytes isolated from spleens and mLNs of WT and *Cyp1a1^-/-^* mice. The lymphocytes were cultured in the presence or absence of UroA under T-cell differentiation conditions. After 3 days, we analyzed the CD4^+^ T cells population of WT and *Cyp1a1^-/-^* mice in the cultures. As shown in **Figure 8A**, UroA treatment significantly down regulated CD4^+^ T cells compared to vehicle treatment in both spleen and mLNs cultures. In contrast, UroA failed to exert its activities on lymphocytes isolated from spleens/mLNs of *Cyp1a1^-/-^* mice (**Figure 8B**). Next, we analyzed the T-reg and Th17 cell population in these cultures. UroA treatment significantly enhanced T-reg population of WT (spleen and mLN) compared to vehicle treatment (**Figures 8C, E**). UroA did not show any impact on Th17 cell population (**Figures 8C, E**). UroA failed to expand T reg cell population in cultures of lymphocytes isolated from *Cyp1a1^-/-^* mice (**Figures 8D, F**). These results highlight the requirement of CYP1A1 for UroA-mediated expansion of T-reg cells, which may potentially be responsible for anti-colitis properties of UroA.

## Discussion

The pathogenesis of IBD is multifactorial resulting from combinations of genetic polymorphism, environmental factors, diet, altered microbiota, and dysregulation of the immune system (43). Current therapies include usage of mesalamine (5-aminosalicylic acid; 5ASA), immune suppressants (anti-TNF-α). However, some patients do not respond to 5ASA, and immune suppressants carry black box warnings including elevated risk of tumors and severe infections (44). Thus far, there are no drugs available that directly target gut barrier dysfunction to restore gut homeostasis. Previously, we and others showed that AhR ligands enhanced gut barrier function through upregulating tight junctional protein (2, 45, 46). Our group demonstrated that treatment with AhR ligand UroA upregulated tight junctional proteins, blocked inflammatory cytokines and mitigated chemical-induced colitis in mouse models in an AhR-dependent manner (11). It is well-established that the activation of AhR leads to an induction of CYP1A1, which is responsible for xenobiotic detoxification. Maturation and activities of intestinal cytochromes P450 enzymes are previously reported to get influenced by intestinal microflora and showed a potential activity beyond drug metabolism (19). Moreover, studies showed that intestinal CYPs abundance and its effect on intestinal ecosystem imparts a protective effect in human gut (20). Although many studies described the role of AhR in regulation of IBD pathogenesis and gut barrier integrity, the functional role of CYP1A1 in IBD pathogenesis is poorly understood. Here, we explored the role of CYP1A1 in UroA-mediated protective activities against DSS-induced colitis using *Cyp1a1^-/-^* mice.

Our results suggested that the treatment with UroA protected the WT mice, but not *Cyp1a1^-/-^* mice from DSS-induced colitis. It is evident from our data that UroA treatment failed to protect from DSS-induced loss of body weight, shortening of colons, colonic inflammation and gut barrier damage in *Cyp1a1^-/-^* mice (**Figure 1**) suggesting the requirement of CYP1A1 for UroA- mediated activities. CYP1A1 is generally known as a xenobiotic/drug metabolizing enzyme that assists in excreting toxic chemicals, but functional role at mucosal sites and gut barrier function is poorly understood. For the first time, our results highlight the importance of CYP1A1 in gut barrier functional activities, where we showed that UroA treatment did not reduce DSS-induced gut permeability, loss of tight junctional proteins (colons) in *Cyp1a1^-/-^* mice compared to WT (**Figure 3**). It is possible that UroA-mediated activation of AhR led to induction of CYP1A1, which may have unknown downstream signaling cascades that may be responsible for enhanced gut barrier function.

Increased neutrophil infiltration at mucosal inflammatory sites contributes to pathogenesis of IBD. As expected, challenging with DSS increased neutrophilic infiltration as it was evident from increased MPO levels and was reduced upon UroA treatment in WT mice, but not in *Cyp1a1^-/-^* mice. Similarly, UroA treatment decreased DSS-induced inflammatory cytokines (TNF-α and IL-6) in WT mice, but not in *Cyp1a1^-/-^* mice. The direct involvement of CYP1A1 in UroA-mediated anti-inflammatory activities is deduced from BMDM experiment, where UroA inhibited LPS-induced TNF-α and IL-6 in WT BMDM, but in *Cyp1a1^-/-^* BMDM. Overall, these studies suggested UroA-mediated its anti-inflammatory through CYP1A1. Previously, we showed that AhR was required for UroA-mediated anti-inflammatory activities (11). It is also possible that UroA metabolization by CYP1A1 may generate active unidentified metabolites that potentially render these beneficial effects. Alternatively, UroA-induced CYP1A1 is directly/indirectly regulating downstream signaling events to mediate UroA physiological functions. It is worthwhile to test this hypothesis in future studies.

Immune abnormalities in colitis mice have been reported. In agreement with previous observations that DSS-treatment caused significant changes in macrophages, DC and CD4^+^ T cell populations both in spleen and mLN. Our findings indicate that UroA treatment reversed the immune abnormalities (restored immune population comparable to control mice) in WT mice, but not in *Cyp1a1^-/-^* mice. It is well-established that increased levels of colonic inflammatory macrophages significantly promote IBD pathogenesis (47, 48). Our results suggested that UroA treatment significantly reduced DSS-induced inflammatory macrophages both in mLN and colonic tissues. Further, it will be interesting to characterize the UroA-mediated macrophage plasticity in DSS-induced colitis. Emerging evidence suggests that intestinal DC play an important role in regulation of immunity in the gut. DCs can acquire gut antigens in the intestinal lumen, and they have the unique ability to regulate the activity of T cells as they present antigen for CD4^+^ or CD8^+^ T cells (49). Depending on the tissue specific location of the DCs, they influence lymphocyte migration and activity. It has been reported that CD11c^+^ DCs reduced the immunological response associated with TNBS induced experimental colitis in BALB/c mice as it regulated the T cell responses (50). Protective effects of DCs in colitis helps to reduce the severity of colitis.

A growing body of evidence suggests that CD4^+^ T cells play a major role in IBD pathogenesis (51, 52). It was reported that CD4^+^ T cells were enriched in inflammatory sites of CD and UC patients, and blocking/depletion of CD4^+^ T cells by CD4 antibody significantly caused remission from IBD (35, 36). Unlike CD4 antibody, UroA treatment did not cause complete loss of CD4^+^ T cells but restored similar to control mice re-establishing homeostasis. Hall et al. showed that the peak number of CD4+ T cells occurred on day 12 after initiation of DSS administration (53). Moreover, they also showed that the changes in the percentage of T cells in spleen and mLNs can occur as early as day 1, indicating that during acute colitis the adaptive immune system is already changing, even though T cells are not required for induction. It was shown in DSS-induced acute colitis mouse model that increased CD4+ T cell population both mLN and inflamed colonic tissues by immuno-PET scanning methods (54). Previous studies suggested that homeostatic level of CD4^+^ T cells are required for host protective responses, however, hyperactivated CD4^+^ T cells contribute to DSS-induced acute colitis pathology (54–58). Here, we showed that DSS-challenged mice displayed increase in the CD4^+^ T cells in mLN both in WT and *Cyp1a1^-/-^* mice. Treatment with UroA significantly reduced/restored CD4^+^ T cell population in WT mice, but not in *Cyp1a1^-/-^* mice suggesting critical role of CYP1A1 in UroA-mediated correction of immune abnormalities in colitis. It will be interesting to evaluate the active status of CD4+ T cells to determine impact of UroA on T cell active status in colitis. Next, we evaluated the impact of UroA on CD8^+^ T cell population (**Supplementary Figure 3**). The percentage of CD8^+^ T cells (CD3^+^CD8^+^) were significantly reduced in WT and *Cyp1a1^−/−^* mice (albeit very low in *Cyp1a1^−/−^* mice) after DSS challenge. Interestingly, UroA treatment restored the CD8^+^ T cells frequencies in WT mice, but failed *Cyp1a1^−/−^* mice suggesting UroA treatment reduced DSS-induced immune abnormalities in CYP1A1-dependent manner. The direct role of UroA-CYP1A1 axis in regulation of immune cell functions in colitis require further investigation.

Next, we evaluated which type of T helper (Th) cells and their associated cytokines are critical for pathogenesis or protection against IBD. Our data suggested that UroA treatment restored the T reg population in DSS-challenged WT mice. It is important to recall that a significant reduction of peripheral T-reg cells has been reported in IBD patients (37) and conversely a variety of studies suggest that increased levels of T-regs may protect against IBD pathogenesis (38–40). Previously, it was shown that AhR promotes T reg expansion in the gut (41, 42), but the role of AhR-CYP1A1-promoted T regs with respect to the control of colitis is not clearly understood. Our studies suggest that treatment with UroA promoted expansion of T regs in DSS-challenged WT mice, but not in *Cyp1a1^-/-^* mice (**Figure 7**). We obtained the similar results in *ex vivo* T cell differential experiments, where UroA treatment resulted in expansion of T reg cells (**Figure 8**). Our unpublished data also suggested that UroA failed to promote T reg expansion in AhR^-/-^ mice suggesting AhR-CYP1A1 axis is critical for UroA mediated T reg expansion. Significant literature is available describing the role of AhR in expansion of T reg cells based on its ligand specificity (41, 59, 60). For example, AhR activation by various ligands such as 2,3,7,8-Tetrachlorodibenzodioxin (TCDD), 2-(1’H-indole-30-carbonyl)-thiazole-4-carboxylic acid methyl ester (ITE), L-kynurenine (L-Kyn), or Laquinimod metabolites increases FoxP3^+^ Treg cells through different mechanisms. These include direct transactivation and the induction of epigenetic modifications that control FoxP3 transcription, and through the modulation of DCs. Additionally, it was shown that AhR was important for T reg cells gut homing and function (41), and AhR-expressing T reg cells have enhanced suppressive activity in a model of colitis (41). The molecular mechanisms responsible for UroA-mediated expansion of T reg cells are yet to identified. It is possible that UroA-induced expansion of the T reg population is dependent on AhR-CYP1A1 axis and may play an important role in UroA-mediated protective activities against colitis. Cell-specific deletion of AhR and CYP1A1 may provide better understanding of how UroA-mediated activities regulate differentially to mitigate colitis.

It is pertinent to recall that Schiering et al. showed that intestinal epithelial cells serve as gatekeepers for the supply of AhR ligands to the host (23). They have demonstrated that deletion of three AhR-controlled CYP enzymes CYP1A1, Cyp1A2 and CYP1B1 resulted in accumulation of AhR ligand, FICZ and enhanced AhR pathway. Further, they observed that lack of these Cyp enzymes increased the frequencies of IL-22-producing cells after 4 days culture under Th17-cell-inducing conditions in the presence of FICZ suggesting increased AhR activity. It is interesting to note that these triple Cyp knockout mice cleared the *Citrobacter rodentium* more effectively than wild type mice. Thus, increased AhR ligand availability in these triple Cyp knockout mice to intestinal immune cells led to increase in IL-22 and promoted resistance to enteric infection (23). It was shown by Kyoreva et al. CYP1A1 enzyme activity influences the skin inflammation *via* regulation of AhR pathway (15). They demonstrated that mice constitutively expressing CYP1A1 displayed exacerbated immune cell activation and drastic reduction of γδ T cells, and skin pathology mirroring AhR^-/-^ mice. Blocking of CYP1A1 ameliorated the skin immunopathology restoring beneficial AhR signaling pathways suggesting blocking CYP1A1 activity may represent an alternative strategy to activate anti-inflammatory function of AhR in skin inflammation (15).

In contrast to above observations, our data suggested that UroA required the expression of CYP1A1 to mediate AhR-dependent beneficial activities. It is possible that the accumulation of AhR ligands in *Cyp1a1^-/-^* mice may not be at the same levels compared to triple Cyp knockout mice (lack of CYP1A1, CYP1A2 and CYP1B1 enzymes) to render the beneficial activities against DSS-induced colitis. This could be attributed to activities of remaining CYP enzymes in *Cyp1a1^-/-^* mice that are responsible for metabolism of other AhR ligands. Another possibility is that CYP1A1 enzyme could potentially metabolize UroA leading generation of active intermediate compounds, which may exert the beneficial activities that were observed upon UroA treatment. Yet another possibility is that selective activation of AhR-CYP1A1 by UroA leads to downstream signaling cascade to reduce inflammation, upregulate tight junction proteins and modulate immune responses. These speculations require further investigation by determining levels of AhR ligands, UroA intermediate metabolites and CYP1A1 downstream signaling cascading including post-translational modifications etc. Further, testing the effects of UroA in CYP1A1-overexpressing mice (R26CYP1A1) (23) will define the role of CYP1A1 in UroA metabolism and its impact on beneficial effects in mitigating colitis or other inflammatory disorders. Thus, overall levels and types of AhR ligands as well as level of cell-specific AhR expression play a pivotal role in gut homeostasis. In this regard, the balance between CYP1A1 and other Cyp enzymes and their potential for activating downstream signaling pathways provide another set of critical regulatory events and feedback loop in maintaining gut homeostasis.

**Figure 9:**
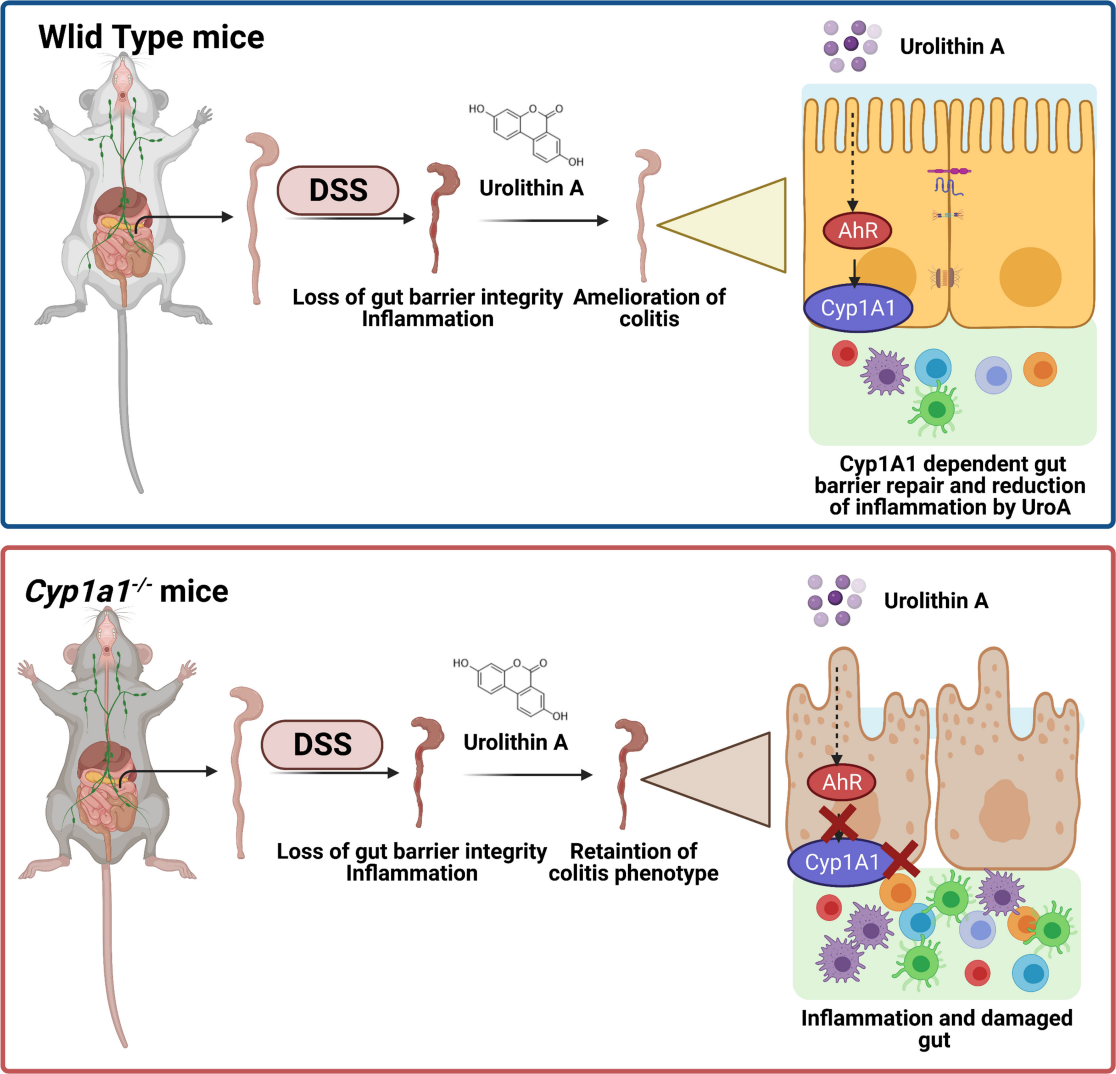
UroA mitigates colitis in CYP1A1-dependent manner. UroA treatment protected wild type mice from DSS-induced colitis, but failed in *Cyp1a1^-/-^* mice.

In summary, we showed that UroA-mediated protective activities against colitis dependent upon expression of CYP1A1 in addition to AhR (**Figure 9**). Importantly, UroA mediated gut barrier functions, anti-inflammatory activities, and T reg expansion depends upon CYP1A1 expression both *in vitro* and *in vivo* models. We propose that CYP1A1 potentially have additional physiological roles in regulation of gut homeostasis in addition to drug metabolism activities. It is also possible that CYP1A1 or other Cyp enzymes metabolic products of UroA may also have important functions, which require further investigation.

## Data availability statement

The raw data supporting the conclusions of this article will be made available by the authors, without undue reservation.

## Ethics statement

The animal study was reviewed and approved by Institutional Animal Care and Use Committee (IACUC), University of Louisville, Louisville, KY, USA.

## Author contributions

SG and VJ designed, performed experiments and written the MS. BH participated in discussions of the study and writing of MS. BM provided Cyp1a1-/- mice and participated in discussions of the study. All authors contributed to the article and approved the submitted version.

## Funding

VJ is supported by NIH/NCI (CA191683), NIH/NIGMS CoBRE grant (P20GM125504-01), P30ES030283 (NIH/NIEHS), The Jewish Heritage Fund for Excellence Research Enhancement Grant and BCC, and BM is in part supported by 5P42 ES027725.

## Acknowledgments

We acknowledge Dr. Uttara Saran for helping during animal euthanization.

## Conflict of interest

VJ is one of the scientific co-founders of Artus Therapeutics LLC.

The remaining authors declare that the research was conducted in the absence of any commercial or financial relationships that could be construed as a potential conflict of interest.

## Publisher’s note

All claims expressed in this article are solely those of the authors and do not necessarily represent those of their affiliated organizations, or those of the publisher, the editors and the reviewers. Any product that may be evaluated in this article, or claim that may be made by its manufacturer, is not guaranteed or endorsed by the publisher.
