# Supplementary material for: Cytochrome P450 1A1 is essential for the microbial metabolite, Urolithin A-mediated protection against colitis
Source: Front Immunol. 2022 Sep 8;13:1004603. doi: 10.3389/fimmu.2022.1004603 (PMC9493474; doi:10.3389/fimmu.2022.1004603)
Supplement: Supplementary file 1 [file DataSheet_1.pdf]

Supplementary Data

Ghosh et al

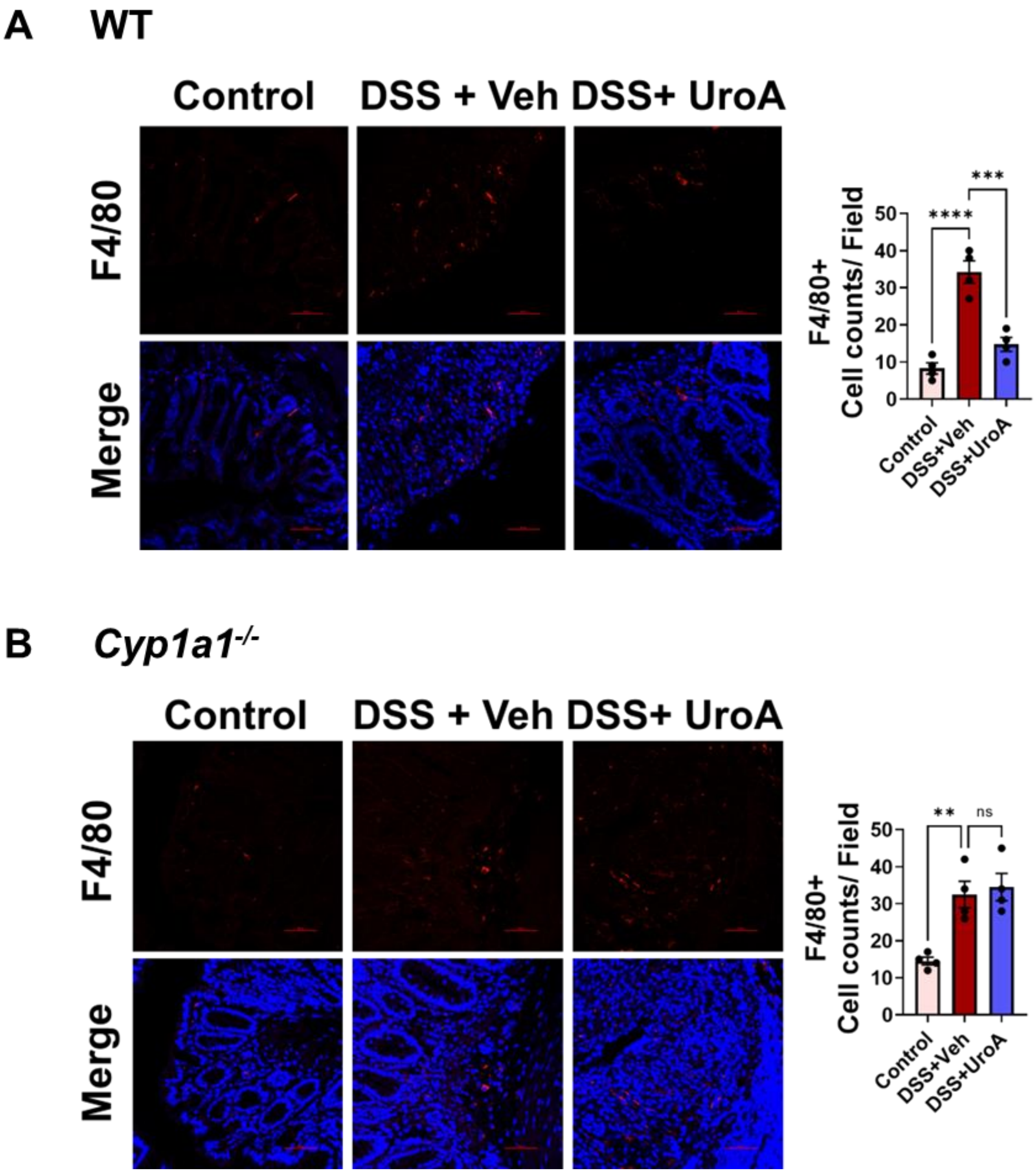

Supplementary Figure 1: UroA treatment reduced the DSS-induced inflammatory macrophages in CYP1A1-dependent manner. Mice were subjected to acute colitis model as described in Figure 1. **A-B.** Colon section of WT and *Cyp1a1*$^{-/-}$ mice (Vehicle, DSS+Veh, DSS+UroA) were stained with anti- F4/80 antibody followed by secondary antibody tagged with Alexa Fluor 594 (Red). The nucleus was stained with DAPI (blue). The fluorescence images were captured using Nikon A1R confocal microscope. The scale bar indicates 50 µm. The number of F4/80^{+} cells per view were counted and plotted. Data are shown as mean ± SEM ***$p < 0.001$; **$p < 0.01$ *$p < 0.05$.

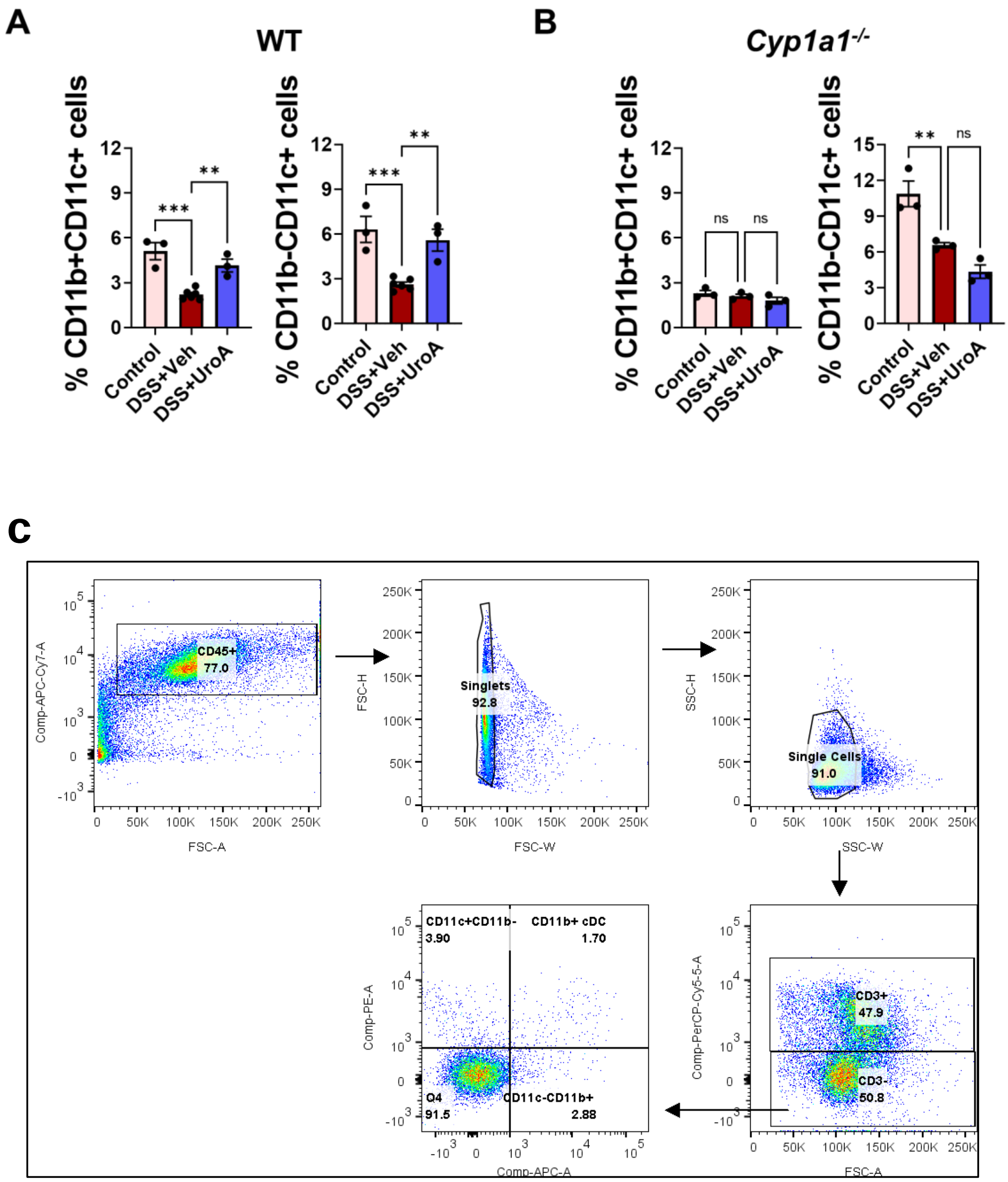

Supplementary Figure 2: UroA regulates CD11c$^+$ cells independent of CD11b in DSS challenged mice. Mice were subjected to acute colitis model as described in Figure 1. Immune cells from WT and *Cyp1a1*$^{-/-}$ mice were analyzed using standard flow cytometric procedures. The percentages of CD11b$^+$CD11c$^+$ and CD11b$^-$CD11c$^+$ cells in the mesenteric lymph node of WT **(A)** and *Cyp1a1*$^{-/-}$ mice **(B***)* are shown. Statistics were performed by One Way ANOVA test using Graphpad Prism 9. ****$p < 0.0001$ ***$p < 0.001$; **$p < 0.01$ *$p < 0.05$; ns: not significant. Error bars, ±SEM. C. Representative gating strategy for CD11b+CD11c+ and Cd11b-CD11c+ populations is shown.

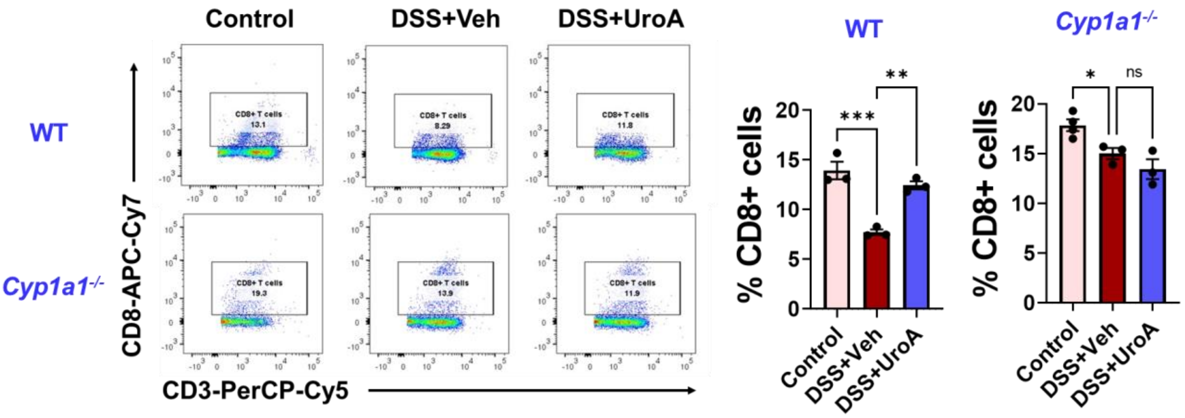

Supplementary Figure 3: UroA fails to restore CD8+ T cells abnormalities in *Cyp1a1*$^{-/-}$ mice. Mice were subjected to acute colitis model as described in Figure 1. Immune cells from WT and *Cyp1a1*$^{-/-}$ mice were analyzed using standard flow cytometric procedures. The percentages of CD8+ T cells in the mesenteric lymph node are shown. Statistics were performed by One Way ANOVA test using Graphpad Prism 9. ****$p < 0.0001$ ***$p < 0.001$; **$p < 0.01$ *$p < 0.05$; ns: not significant. Error bars, ±SEM.

Supplementary Table 1: List of antibodies used in western blot -

**Antibody**	**Company**	**Catalogue number**	**Dilution**
ZO-1 Antibody	ProteinTech	A21773-1-AP	1:1000
Claudin-4 Antibody (A-12) HRP	SCBT	sc-376643 HRP	1:500
Occludin Rabbit Polyclonal antibody	ProteinTech	3409-1-AP	1:1000
HRP-Conjugated Beta Actin Antibody	ProteinTech	RP-60008	1:5000
Goat anti-mouse IgG (H+L), HRP conjugate	ProteinTech	SA00001-1	1:5000
Goat anti-rabbit IgG (H+L), HRP conjugate	ProteinTech	SA00001-2	1:5000

Supplementary Table 2: List of antibodies used in Flow cytometry and immunostaining -

**Antibody**	**Company**	**Catalogue number**
PerCP/Cyanine5.5 anti-mouse CD3ε Antibody	BioLegend	100327
PE/Cyanine7 anti-mouse CD4 Antibody	BioLegend	100421
APC anti-mouse/human CD11b Antibody	BioLegend	101211
PE anti-mouse F4/80 Recombinant Antibody	BioLegend	157304
APC anti-mouse CD8a Antibody	BioLegend	100711
APC/Cyanine7 anti-mouse CD45 Antibody	BioLegend	103115
PE anti-mouse CD11c Antibody	BioLegend	117307
APC/Cyanine7 anti-mouse CD4 Antibody	BioLegend	100413
PE/Cyanine7 anti-mouse CD25 Antibody	BioLegend	102015
APC/Cyanine7 anti-mouse CD25 Antibody	BioLegend	101917
PE anti-mouse FOXP3 Antibody	BioLegend	126403
Anti-Foxp3 Antibody (mouse), APC, clone 3G3	Sigma-Aldrich	MABF730
FITC anti-mouse IL-17A Antibody	BioLegend	506907
FOXP3 Rabbit anti-Human, Mouse	Invitrogen	PI700914
Goat anti-Rabbit IgG (H+L) Highly Cross-Adsorbed Secondary Antibody, Alexa Fluor™ 647 -	Invitrogen	A-21245
